# Dietary intervention for adult survivors of cancers other than breast cancer: A systematic review

**DOI:** 10.1097/MD.0000000000038675

**Published:** 2024-06-28

**Authors:** Hideo Matsumoto, Seiji Onogawa, Norihiro Sonoi, Masano Sagawa, Shigeki Wakiyama, Ryo Ogawa, Yasuhiro Miyazaki, Shigeyuki Nagata, Takehiro Okabayashi, Susumu Tazuma, Akihiko Futamura, Yu Uneno, Naoki Higashibeppu, Joji Kotani

**Affiliations:** a Department of Surgery, Mitsugi General Hospital, Onomichi, Hiroshima, Japan; b Department of Internal Medicine, JA Onomichi General Hospital, Onomichi, Hiroshima, Japan; c Center for Education in Medicine and Health Sciences, Okayama University Graduate School of Medicine, Dentistry and Pharmaceutical Sciences, Okayama, Japan; d Department of Surgery, Tokyo Women’s Medical University Adachi Medical Center, Tokyo, Japan; e Department of Surgery, Machida Municipal Hospital, Machida, Tokyo, Japan; f Department of Gastroenterological Surgery, Nagoya City University Graduate School of Medical Sciences, Nagoya, Aichi, Japan; g Department of Gastroenterological Surgery, Osaka General Medical Center, Osaka, Japan; h Department of Surgery, Hiroshima Red Cross Hospital and Atomic-Bomb Survivors’ Hospital, Hiroshima, Japan; i Department of Gastroenterological Surgery, Kochi Health Sciences Center, Kouchi, Japan; j Department of Pharmacy, Fujita Health University Nanakuri Memorial Hospital, Tsu, Mie, Japan; k Department of Therapeutic Oncology, Graduate School of Medicine, Kyoto University, Kyoto, Japan; l Department of Anesthesia and Critical Care, Kobe City Medical Center Hospital, Kobe, Hyogo, Japan; m Division of Disaster and Emergency Medicine, Department of Surgery Related Kobe University Graduate School of Medicine, Kobe, Hyogo, Japan.

**Keywords:** cancer survivors, dietary intervention, undernutrition, systematic review, quality of life

## Abstract

**Introduction:**

Healthy eating and weight control are recommended for cancer survivors; however, dietary interventions are not routinely offered to them. This study aimed to assess the effects of dietary interventions on survival, nutritional status, morbidity, dietary changes, health-related quality of life (QOL), and clinical measures in cancer survivors.

**Methods:**

Searches were conducted from October 1, 2018 to November 21, 2011 in the Medline, EMBASE, CENTRAL, Emcare, and DARE electronic databases. We included randomized controlled trials (RCTs) that involved individuals diagnosed with cancer, excluding conference abstracts, case studies, other reviews, and meta-analyses, and screened the articles.

**Results:**

Eight studies were included in this meta-analysis. We observed significant improvements in QOL and clinical data in 3 of 6 studies and in one study, respectively, significant weight loss on anthropometry in 2 of 5 studies, and dietary improvement in 4 of 5 studies of adult cancer survivors. However, we did not observe any benefits of dietary intervention for cancer survivors with undernutrition.

**Discussion:**

Dietary interventions for adult cancer survivors might contribute to improving their nutritional status; however, further clarification requires a study that standardizes the intervention method. Furthermore, RCTs are required to determine the effects on cancer survivors with undernutrition.

## 1. Introduction

There were approximately 19.3 million new cases of cancer and almost 10 million related deaths worldwide in 2020.^[1]^ More than 16.9 million Americans with a history of cancer were alive on January 1, 2019; this number is projected to reach more than 22.1 million by January 1, 2030 based on population aging and growth.^[2]^ In Japan, according to statistics from the National Cancer Research Center, the 5-year relative survival rate for patients diagnosed with cancer between 2009 and 2011 was 64.1%, and this rate has increased since the start of the statistics.^[3]^ Therefore, there is an increase in the number of cancer survivors. However, it should be noted that cancer survivors not only have a higher risk of secondary cancer^[3–6]^ but also a higher incidence of comorbidities such as heart attack, stroke, or obesity^[7,8]^ compared with the general population.

On the other hand, 999,000 people were diagnosed with cancer in 2019 in Japan, with the most common diagnosis being colorectal cancer, followed by lung, stomach, breast (97,812 diagnosed patients), pancreatic, and liver cancers; the 5-year survival rates were 71.4%, 34.9%, 66.6%, 92.3%, 8.5%, and 35.8%, respectively (https://ganjoho.jp/reg_stat/statistics/stat/summary.html). Thus, breast cancer clearly has a better prognosis.

In Japan, gastroenterological and lung cancer account for 53.2% of the total cancer diagnosis, and survivor of such cancer may develop undernutrition after completion of treatment. International dietary recommendation includes diet for health and/or weight control in patients with cancer.^[9–11]^ In 2019, Burden et al conducted a meta-analysis of dietary interventions among adult cancer survivors using Cochrane Library data.^[12]^ In this analysis, the primary outcome (mortality) and many secondary outcomes, except dietary preferences and body mass index (BMI), were rated as low-certainty evidence. Efforts were mainly aimed at improving nutrition and lifestyle. Nevertheless, in the context of gastrointestinal cancer survivors who develop undernutrition, dietary intervention for such patients may improve their survival rate and quality of life (QOL).

To answer this question, the Japanese Society of Clinical Metabolism and Nutrition set up a guideline development committee that established a Problem/Interventions/Comparisons/Outcome according to the Minds clinical practice guideline development manual 2020 ver. 3.0 (https://minds.jcqhc.or.jp/s/manual_2020_3_0) and conducted systematic review in addition to a previous Cochrane review by our systematic review group.^[12]^ In this review, we aimed to verify the effects of nutritional interventions, especially for patients with undernutrition. Because breast cancer is strongly associated with obesity^[13]^ and its prognosis and quality of life are different from those of other cancers, studies on breast cancer was excluded from this review. This study was conducted as a clinical question in the Japanese Society for Clinical Nutrition and Metabolism guideline development process.

## 2. Methods

### 2.1. Electronic searches

Searches were conducted from October 1, 2018 to November 21, 2021 following the methods of a previous Cochrane review.^[12]^ This review was prepared and conducted/reported according to the recommendations set out in the Minds Manual for Guideline Development 2020 version 3.0 (https://minds.jcqhc.or.jp/s/manual_2020_3_0). Medline, EMBASE, CENTRAL, Emcare, and DARE were searched according to the Minds Manual for Guideline Development (Supplementary 1, Supplemental Digital Content, http://links.lww.com/MD/N34).

### 2.2. Assessment of risk of bias in included studies

Eight authors assessed the titles and full-text articles, extracted the data, and assessed the risk of bias independently and randomly paired in each part. The Grading of Recommendations Assessment, Development, and Evaluation approach was used to rate the certainty of evidence,^[14]^ considering limitations, indirectness, inconsistencies, imprecision, and bias using RevMan 5 (Supplementary 2, Supplemental Digital Content, http://links.lww.com/MD/N35).^[15]^

### 2.3. Types of participants

Participants of the included studies were adult survivors of cancers other than breast cancer who had completed all active anticancer interventions, such as surgery, radiotherapy, chemotherapy, hormone therapy, and molecular targeted drugs. Studies with ≥67% of participants being patients aged 18 years or older were eligible; however, those that reported >60% of participants being patients with breast cancer were excluded.

### 2.4. Types of studies

We included randomized controlled trials (RCTs) involving individuals diagnosed with cancer, excluding conference abstracts, case studies, other reviews, and meta-analyses. We screened the articles using the Rayyan (https://www.rayyan.ai/) system.

### 2.5. Types of interventions

The interventions included any dietary advice provided by any method, including group sessions, telephone instruction, written materials, or a web-based approach, with or without exercise, according to a previous Cochrane review. The control group did not receive any intervention or normal nutritional guidance.

### 2.6. Types of outcome measures

The measured outcomes included QOL, overall survival, adverse events, anthropometry, secondary malignancies, dietary changes, and clinical data.

The review was submitted to the PROSPERO database (Ref: CRD42022363095) before initiation of the search and can be found at http://www.crd.york.ac.uk/PROSPERO/. This review was prepared in compliance with Preferred Reporting Items for Systematic Reviews and Meta-Analyses (PRISMA) 2020 (https://training.cochrane.org/online-learning/core-software/revman/revman-5-download) (Appendix 3).^[16]^

Based on the national ethical guidelines for medical and health research involving human subjects in Japan, the present systematic review is outside of the scope to be reviewed by local ethical committees.

## 3. Results

**Figure 1.**
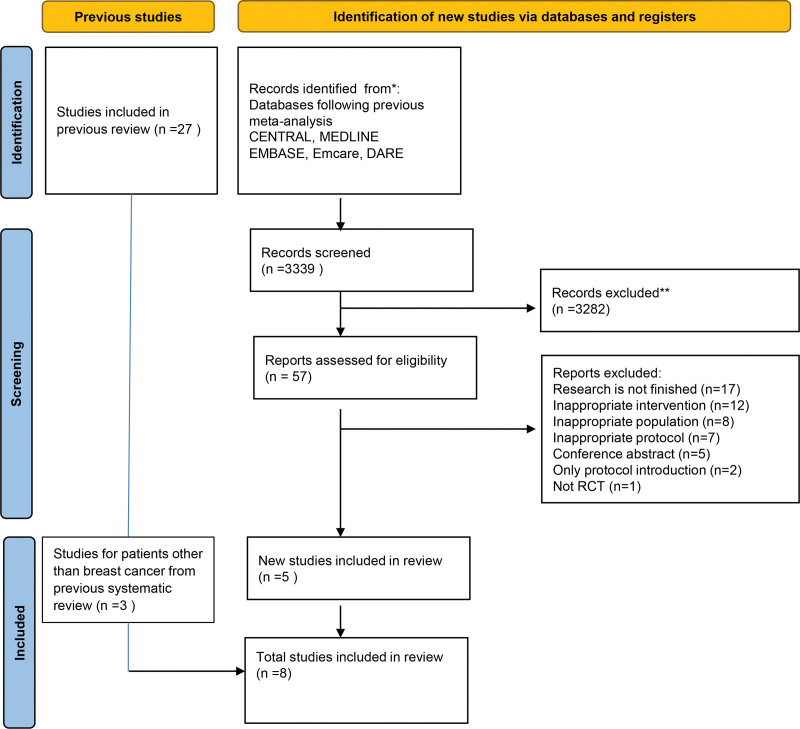
PRISMA flow diagram.

In addition to 27 records from previous Cochrane studies, 3399 records were extracted for this study using the same method, resulting in a total of 60 records after screening: 3 of 27 from previous Cochran studies and 57 of 3399 from this screening. Sixty full-text articles were screened, and after applying exclusion criteria, 8 studies were considered eligible for this study (Fig. 1). Of the 8 studies, 3 were on colorectal cancer, 2 on uterine cancer, 1 on ovarian cancer, 1 on head and neck cancer, and 1 on colon cancer.

The studies reported various intervention method and were broadly divided into 2 categories: 6 studies that included nutrition management and exercise at the same time and 2 studies that only included dietary counseling. Dietary interventions ranged from home-based interventions to residential programs and from regular telephone-based interventions to simultaneous interventions via the web. The timing of study evaluations also varied from 3 months to 2 years (Table 1).^[15, 17–23]^

**Table 1:** Baseline characteristics of the included studies.

Author, year, (country)	Participants/follow up	Outcome	Intervention method/occupation involved	Intervention
Koutoukidis et al 2019 (UK)^[17]^	Adult endometrial cancer survivor within 3 yr of diagnosis Stages I to IVA (n = 60)/estimated at 8 to 24 wk	Diet, physical activity, anthropometry, body composition, hand grip strength, blood pressure, QOL	Shape-up following cancer treatment program: weekly 1.5-h sessions on healthy eating and physical activity based on social cognitive theory and control theory/dietitian	Intervention (n = 24) vs usual care (n = 25)
von Grunigen et al 2012 (Canada)^[15]^	Endometrial cancer survivor within 3 years of diagnosis BMI ≧ 25 (over weight/obese) Stage I or II (n = 75)/estimated at 3, 6, 12 mo	Primary: weight change, secondary: fruit/vegetable servings/d, physical activity	A life style intervention consisting of nutrition, exercise and behavioral modification counseling via telephone, email, newsletter/physician, psychologist, registered dietitian, physical therapist	Intervention (n = 41) vs usual care (n = 34)
Paxton et al 2012 (USA)^[18]^	Ovarian cancer survivor Stage ≧ IIA no evidence of recurrent or progressive disease, complete ≧ 21 yr old treatment ≧ 6 mo BMI ≧ 19.5 (n = 52)/estimated at 6 mo	Weight change, waist to hip ratio, serum albumin, CA-125 dietary intake	Self-monitoring logs and telephone counseling calls/registered dietitian	Intervention (n = 19) vs low fat, high fiber (n = 19)
Kristensen et al 2020 (Denmark)^[19]^	Head and neck cancer survivor treated radiation 1 to 5 year before survey (n = 71)/estimated 3 mo	Weight change, physical function, QOL,	Multidisciplinary residential nutritional rehabilitation program/clinical dietitian, occupational therapist, physiotherapist, psychologist, speech pathologist, physician	Intervention (n = 36) vs usual care (n = 35)
Bourke et al 2011 (UK)^[20]^	Colon cancer survivor (n = 18)/estimated 12 wk	Exercise, dietary behavior, fatigue, QOL, aerobic exercise tolerance, functional capacity, muscle strength, anthropometry	A 12-wk life style intervention supervised and home-based exercise sessions and dietary advice/exercise physiologist	Intervention (n = 8) vs usual care (n = 9)
Hawkes et al 2013 (Australia)^[21]^	Colorectal cancer survivor diagnosed within previous 12 mo, no metastatic disease (n = 410)/estimated 12 mo	Primary: physical activity, QOL, cancer-related fatigue, secondary: BMI, dietary intake	An 11-mo telephone delivered health coaching, handbook, postcard promote, pedometer, quarterly study newsletter/University degrees in nursing, psychology, or health promotion	Intervention (n = 159) vs usual care (n = 163)
Ho et al 2020 (Hong Kong)^[22]^	Colorectal cancer survivor diagnosed within 1 year completion of primary treatment (n = 224)/estimated at 6, 12, 18, 24 mo	QOL	Dietary: face-to-face motivational interviews (2 sessions,), motivational phone calls every 2 wk, physical activity: face-to-face motivational interviews (1 session) motivational phone call 2 wk/dietitians, trained research assistant	Dietary + physical activity (n = 55), dietary only (n = 56), physical activity only (n = 56), and usual care (n = 56)
Van Blarigan et al 2020 (USA)^[23]^	Colon or rectal cancer survivor not actively undergoing chemotherapy considered disease-free or stable disease (n = 50)/estimated at 0, 12, 24 wk	Feasibility and acceptability dietary assessment	The website included goal setting, daily tracking of target food groups, visual summaries of tracked dietary intake and progress toward goals, recommendations, recipes, meal planning, frequently asked questions, and a profile page	Intervention (n = 22) vs usual care (n = 20)

BMI = body mass index, QOL = quality of life.

A qualitative systematic review was performed, and no meta-analysis was performed because of differences in intervention methods and target cancer types.

### 3.1. Quality of life

QOL was discussed in 6 of 8 studies. Intervention was significantly beneficial in 3 of the 6 studies. Of the 3 studies, 2 used the European Organization for Research and Treatment of Cancer Quality of Life Questionnaire for patients with uterine cancer and head and neck cancer, and 1 used the Short-Form-12 questionnaire for patients with colorectal cancer. In the intervention group, the global QOL significantly improved at 24 weeks in patients with ovarian cancer, and the QOL related to daily role function (*P* = .041), pain (*P* = .048), and speech problem (*P* = .040) improved in patients with head and neck cancer (Table 2).^[19]^ Systemic and cancer-specific QOL significantly improved in patients with colorectal cancer. The remaining 3 studies that showed no significant benefit from intervention used rigorous dietary and exercise interventions.

**Table 2:** Outcome of included study.

Author, year, (country)	Anthropometry	QOL assessment method/result	Evaluation method/dietary change	Clinical data
Koutoukidis et al 2019 (UK)^[17]^	Weight reduction at 8 wk (*P* = .007) but no change at 24 wk, body composition: no significant difference	EORTC QLQ-C30/global quality of life significantly improved in intervention at 24 wk	Alternative Healthy Eating Index 2010, at 8 wk intervention group was improved compared to usual care 7.5	NE
von Grunigen et al 2012 (Canada)^[15]^	Weight: −4 kg at 8 mo (*P* < .01) −4.6 kg at 12 mo (*P* < .01)	NE	Nutrition Data System for Research Software (NDSR) v.2008 and 2009/−217.8 kcal at 6 mo (*P* < .001) −187.2 kcal at 12 mo (*P* < .001)	NE
Paxton et al 2012 (USA)^[18]^	Weight: no significant difference	SF-36/no significant difference	Nutrition Data System for Research(Nutrition Coordinal Center, University of Minnesota, Minneapolis MN)/fiber intake, daily servings of juice, vegetables were significantly improved	Significantly increased albumin, lutein, zeaxanthin, retinol, retinol palmitate levels
Kristensen et al 2020 (Denmark)^[19]^	Weight: no significant difference	EORTC QLQ-C30/improved in “role function” (*P* = .041), speech problem (*P* = .040), and pain (*P* = .048) in the intervention group	NE	NE
Bourke et al 2011 (UK)^[20]^	Weight, BMI: No significant difference − waist to hip ratio significantly improved (*P* = .02)	FACT-F, FACT-C/no significant difference	Fiber intake was improved in intervention group (*P* = .44)	NE
Hawkes et al 2013 (Australia)^[21]^	BMI: significantly decreased at 12 mo (−0.9 kg/m^2^, *P = *.001).	SF-36, cancer-related fatigue/no significantly difference	Cancer Council Victoria, Food Frequency Questionnaire/no significant difference	NE
Ho et al 2020 (Hong Kong)^[22]^	NE	FACT-C, FACT-G, SF-12, improved significantly in the generic and cancer-specific QOL, and depression	NE	NE
Van Blarigan et al 2020 (USA) ^[23]^	NE	NE	HIPPA-complaint secure server/higher intake of whole grain	NE

BMI = body mass index, EORTC QLQ 30 = Questionnaire developed to assess the quality of life of cancer patients, FACT-C = The Functional Assessment of Cancer Therapy – Colorectal Scale, FACT-F = The Functional Assessment of Chronic Illness Therapy – Fatigue, FACT-G = The Functional Assessment of Cancer Therapy – general score, NE = not evaluated, QOL = quality of life, SF-12 = Short-forum 12, SF-36 = Short-Form 36.

### 3.2. Anthropometry

Body weight was measured in 5 studies, and 2 studies found a significant difference in weight loss between the intervention and control groups. Both studies were on endometrial cancer. In the first study, there was weight loss at 8 weeks (*P* = .007) but no difference at 24 weeks.^[17]^ The other study showed a weight loss of −4 kg in the 8th month (*P* < .01) and −4.6 kg in the 12th month (*P* < .01).^[15]^ BMI was measured in 1 study; it decreased significantly in the intervention group (−0.9 kg/m^2^, *P* = .01) in patients with colon cancer (Table 2).^[21]^

### 3.3. Dietary changes

Dietary improvement was assessed in 6 RCTs, and significant improvements were observed in 5 RCTs. The evaluation methods varied, with 1 trial reporting a significant decrease in calorie intake in patients with endometrial cancer (*P* < .01)^[20]^, 2 showing an increase in fiber intake in patients with ovarian cancer^[21]^ and those with colon cancer,^[20]^ 1 showing an increase in grain intake with colon cancer,^[23]^ and 1 showing an improvement in food intake with colon and rectal cancer (Table 2).^[21]^

### 3.4. Clinical data

One study reported on intervention for patients with ovarian cancer. The intervention involved the use of self-monitoring and primarily telephone consultation to promote fruit and vegetable intake, leading to significantly increased albumin, zeaxanthin, lutein, and retinol levels (Table 2).^[18]^

### 3.5. Overall survival, adverse events, and secondary malignancy

None of the studies reported overall survival rates, adverse events, or secondary malignancies. Furthermore, since the details and influence of undernutrition in adult cancer survivors were not shown, the effect of dietary intervention for these groups was unclear.

## 4. Discussion

In this study, we aimed to verify the effects of nutritional interventions, especially for patients with undernutrition. We observed improvements in QOL and clinical data, weight loss on anthropometry, and nutrition in adult cancer survivors in the intervention group. However, we could not prove the benefits of dietary interventions for cancer survivors with undernutrition.

In a previous systematic review, Burden et al reported that the dietary intervention and control groups showed little or no difference in the risk of mortality (hazard ratio [HR], 0.98, 95% confidence interval [CI] 0.77–1.23; 1 study; 3107 participants; low-certainty evidence) or secondary malignancies (HR 0.99, 95% CI 0.84–1.15; 1 study; 3107 participants; low-certainty evidence).^[13]^ Comorbidities were not measured. Subsequent outcomes reported after 12 months showed little or no difference between the intervention and control groups in energy intake at 12 months (mean difference [MD] −59.13 kcal, 95% CI −159.05 to 37.79; 5 studies: 3283 participants; moderate-certainty evidence). In addition, compared with the control group, the dietary intervention group showed slight increases in fruit and vegetable servings (MD 0.41 servings, 95% CI 0.10–0.71; 5 studies; 834 participants; moderate-certainty evidence); mixed results for total fiber intake (MD 5.12 g, 95% CI 0.66–10.9; 2 studies; 3127 participants; very low-certainty evidence); and possible improvement in Diet Quality Index (MD 3.46, 95% CI 1.54–5.38; 747 participants; moderate-certainty evidence). For anthropometry, the dietary intervention group showed a slight decrease in BMI (MD −0.79 kg/m^2^, 95% CI −1.50 to −0.07; 4 studies; 777 participants; moderate-certainty evidence) compared with the control group. For QOL, there were mixed results; different quality assessment tools were used, and the evidence was of low to very low certainty. No adverse events were reported in any of the studies. The Cochrane review consisted of 27 studies, and 17 of them were on patients with breast cancer. Seven of the remaining 10 studies included patients with breast cancer, accounting for ≥50% of the study population. Only 3 studies from the previous Cochrane review were included in our study.

We started this study to prove the efficacy of nutritional intervention for cancer survivors, especially patients with undernutrition; thus, we excluded patients with breast cancer because of the strong association between breast cancer and obesity^[13]^ and the behavior breast cancer differs from other cancer types in terms of progress and prognosis. However, there have been few studies on dietary interventions for survivors of cancers other than breast cancer, and the intervention methods, target cancer types, and evaluation methods were inconsistent, making a meta-analysis impossible. Hence, our results could not prove the benefits of dietary intervention for cancer survivors with undernutrition. The purpose of our study is slightly different from that of the previous Cochrane review; and it might have been easier to understand if patients with undernutrition were targeted from the beginning instead of continuing the previous Cochrane review. However, this study reveals for the first time the lack of studies of nutritional interventions for cancer survivors with undernutrition.

This study had the following limitations. All RCTs were small-scale and reported distinctive intervention methods, intervention periods, and evaluation methods. Since the intervention and evaluation periods were short, the survival rate and incidence of secondary cancers could not be evaluated. In addition, adverse events are unlikely to occur during dietary intervention and exercise therapy. Four studies showed an increase in physical activity levels, and this should be included as one of the outcomes. A long-term observational RCT involving various cancer types, preferably with a unified intervention method, is necessary to produce results to answer this clinical question.

In conclusion, in this systematic review, no clinical trials were conducted to examine the benefits of dietary intervention for cancer survivors with undernutrition. Therefore, new clinical trials are necessary to examine the benefits of nutritional intervention for undernutrition after gastrointestinal surgery.

## Acknowledgments

We would like to express our deep gratitude to Prof Toshihiro Hirai for his advice regarding this article. The authors are grateful to the members of the Guideline Development Committee, the Board of Directors and the secretariat of the Japanese Society for Clinical Nutrition and Metabolism for their kind support of this review. We also appreciate Dr Norio Watanabe and Cochrane Japan’s support in conducting a systematic literature search. We would like to thank Editage (www.editage.com) for English language editing.

## Author contributions

**Data curation:** Hideo Matsumoto, Seiji Onogawa, Norihiro Sonoi, Masano Sagawa, Shigeki Wakiyama, Ryo Ogawa, Yasuhiro Miyazaki, Shigeyuki Nagata.

**Formal analysis:** Hideo Matsumoto, Seiji Onogawa, Norihiro Sonoi, Masano Sagawa, Shigeki Wakiyama, Ryo Ogawa, Yasuhiro Miyazaki, Shigeyuki Nagata.

**Investigation:** Hideo Matsumoto, Norihiro Sonoi, Masano Sagawa, Shigeki Wakiyama, Ryo Ogawa, Yasuhiro Miyazaki, Shigeyuki Nagata.

**Writing – original draft:** Hideo Matsumoto.

**Writing – review & editing:** Hideo Matsumoto.

**Conceptualization:** Takehiro Okabayashi, Akihiko Futamura, Naoki Higashibeppu, Joji Kotani.

**Methodology:** Takehiro Okabayashi, Akihiko Futamura, Yu Uneno, Naoki Higashibeppu.

**Validation:** Susumu Tazuma.

**Supervision:** Akihiko Futamura, Yu Uneno, Naoki Higashibeppu, Joji Kotani.

**Visualization:** Yu Uneno, Naoki Higashibeppu.

**Project administration:** Joji Kotani.

## Supplementary Material




