# Supplementary material for: Dietary intervention for adult survivors of cancers other than breast cancer: A systematic review
Source: Medicine (Baltimore). 2024 Jun 28;103(26):e38675. doi: 10.1097/MD.0000000000038675 (PMC11466153; doi:10.1097/MD.0000000000038675)
Supplement: Supplementary file 1 [file medi-103-e38675-s001.docx]

**Search strategy**

**CENTRAL search strategy**

#1 MeSH descriptor: [Neoplasms] explode all trees

#2 (cancer* or tumor* or tumour* or neoplas* or malignan* or carcinoma* or adenocarcinoma* or choriocarcinoma* or leukemia* or leukaemia* or metastat* or sarcoma* or teratoma*):ti,ab

#3 #1 or #2

#4 MeSH descriptor: [Diet] explode all trees

#5 MeSH descriptor: [Nutrition Assessment] explode all trees

#6 MeSH descriptor: [Nutrition Therapy] explode all trees

#7 MeSH descriptor: [Nutrition Disorders] explode all trees

#8 MeSH descriptor: [Food Habits] explode all trees

#9 MeSH descriptor: [Food Preferences] explode all trees

#10 MeSH descriptor: [Food] explode all trees

#11 (diet* or nutrition* or nutrient* or food* or feed* or eat* or drink*):ti,ab

#12 (fat* or carbohydrate* or protein* or fruit* or vegetable* or fibre* or fiber* or fish* or meat* or poultry or dairy or salt* or sugar* or cereal* or nut* or seed* or alcohol* or caGeine):ti

#13 (macrobiotic or ketogenic or vegetarian or (low adj (glycemic* or glycaemic*))):ti

#14 #5 or #6 or #7 or #8 or #9 or #10 or #11 or #12 or #13 [#4 is missing here, a mistake of the authors!?]

#15 MeSH descriptor: [Survivors] explode all trees

#16 (survivor* or survival*):ti,ab

#17 #15 or #16

#18 #3 and #14 and #17

**MEDLINE Ovid search strategy**

(exp neoplasms/ OR (cancer* or tumor* or tumour* or neoplas* or malignan* or carcinoma* or adenocarcinoma* or choriocarcinoma* or leukemia* or leukaemia* or metastat* or sarcoma* or teratoma*).ti,ab.) AND (exp diet/ or exp nutrition assessment/ or exp nutrition therapy/ or exp nutrition disorders/ or exp food habits/ or food preferences/ or exp food/ or (diet* or nutrition* or nutrient* or food* or feed* or eat* or drink*).ti,ab. or (fat* or carbohydrate* or protein* or fruit* or vegetable* or fibre* or fiber* or fish* or meat* or poultry or dairy or salt* or sugar* or cereal* or nut* or seed* or alcohol* or caGeine).ti. or (macrobiotic or ketogenic or vegetarian or (low adj (glycemic* or glycaemic*))).ti.) AND (survivors/ OR (survivor* or survival*).ti,ab.) AND (randomized controlled trial.pt. or controlled clinical trial.pt. or randomized.ab. or placebo.ab. or clinical trials as topic.sh. or randomly.ab. or trial.ti.) NOT (exp animals/ not humans.sh.)

**Embase search strategy**

(exp neoplasm/ OR (cancer* or tumor* or tumour* or neoplas* or malignan* or carcinoma* or adenocarcinoma* or choriocarcinoma* or leukemia* or leukaemia* or metastat* or sarcoma* or teratoma*).ti,ab.) AND (exp nutrition/ or exp nutritional disorder/ or (diet* or nutrition* or nutrient* or food* or feed* or eat* or drink*).ti,ab. or (fat* or carbohydrate* or protein* or fruit* or vegetable* or fibre* or fiber* or fish* or meat* or poultry or dairy or salt* or sugar* or cereal* or nut* or seed* or alcohol* or caGeine).ti. or (macrobiotic or ketogenic or vegetarian or (low adj (glycemic* or glycaemic*))).ti.) AND (cancer survivor/ OR (survivor* or survival*).ti,ab.) AND (crossover procedure/ or double-blind procedure/ or randomized controlled trial/ or single-blind procedure/ or random*.mp. or factorial*.mp. or (crossover* or cross over* or cross-over*).mp. or placebo*.mp. or (double* adj blind*).mp. or (singl* adj blind*).mp. or assign*.mp. or allocat*.mp. or volunteer*.mp.)

**Emcare search strategy**

(exp neoplasm/ OR (cancer* or tumor* or tumour* or neoplas* or malignan* or carcinoma* or adenocarcinoma* or choriocarcinoma* or leukemia* or leukaemia* or metastat* or sarcoma* or teratoma*).ti,ab.) AND (exp nutrition/ or exp nutritional disorder/ or (diet* or nutrition* or nutrient* or food* or feed* or eat* or drink*).ti,ab. or (fat* or carbohydrate* or protein* or fruit* or vegetable* or fibre* or fiber* or fish* or meat* or poultry or dairy or salt* or sugar* or cereal* or nut* or seed* or alcohol* or caGeine).ti. or (macrobiotic or ketogenic or vegetarian or (low adj (glycemic* or glycaemic*))).ti.) AND (cancer survivor/ OR (survivor* or survival*).ti,ab.) AND (crossover procedure/ or double-blind procedure/ or randomized controlled trial/ or single-blind procedure/ or random*.mp. or factorial*.mp. or (crossover* or cross over* or cross-over*).mp. or placebo*.mp. or (double* adj blind*).mp. or (singl* adj blind*).mp. or assign*.mp. or allocat*.mp. or volunteer*.mp.)

**DARE search strategy**

**DARE - The Database of Abstracts of Reviews of Effects (DARE) - NHS Centre for Reviews and Dissemination (CRD)**

<https://www.crd.york.ac.uk/CRDWeb/>

Advanced search option

Cancer and|

Survivorship and

Diet and nutrition [*and* should be *or*]

All fields searched MeSH Terms
