# Supplementary figures and images for: Dietary intervention for adult survivors of cancers other than breast cancer: A systematic review
Source: Medicine (Baltimore). 2024 Jun 28;103(26):e38675. doi: 10.1097/MD.0000000000038675 (PMC11466153; doi:10.1097/MD.0000000000038675)

## Slide 1
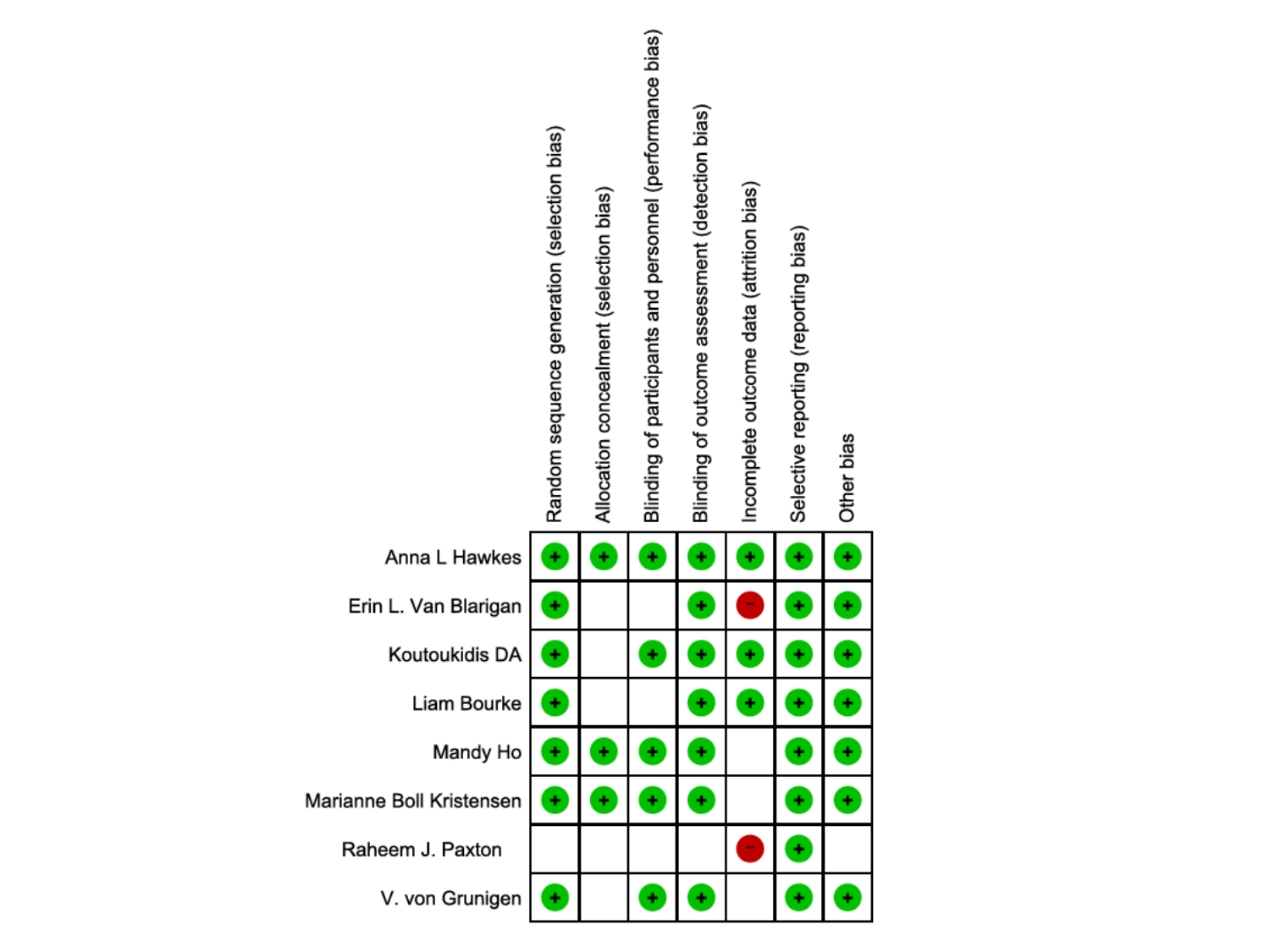

Supplement: Supplementary file 2 [file medi-103-e38675-s002.pptx]
